# Post partum haemorrhage secondary to uterine atony, complicated by platelet storage pool disease and partial placenta diffusa: a case report

**DOI:** 10.1186/1757-1626-1-393

**Published:** 2008-12-13

**Authors:** Shimma S Rahman, Jenny E Myers, Joanna C Gillham, Richard Fitzmaurice, Tracey A Johnston

**Affiliations:** 1 Department of Obstetrics & Gynaecology, St Mary's Hospital, Manchester, UK; 2 Department of Histopathology, Manchester Royal Infirmary, Manchester, UK; 3 Department of Obstetrics & Gynaecology, Birmingham Women's Hospital, Birmingham, UK

## Abstract

**Introduction:**

Uterine atony is the most common cause of primary post partum haemorrhage. We report a case where this was complicated by two rare conditions, platelet storage pool disease and placenta diffusa. Platelet storage pool disease is a platelet aggregation disorder associated with mild to moderate bleeding diathesis. There are limited cases reported in pregnancy. Placenta diffusa is a rare anomaly where all or part of the fetal membranes remain covered by chorionic villi, and is associated with post partum haemorrhage.

**Case presentation:**

A 37-year-old woman was referred to the obstetric haematology clinic for prenatal counselling with a history of three severe post partum haemorrhages, two of which were complicated by placental retention. Platelet aggregation studies confirmed a diagnosis of platelet storage pool disease. She was counselled regarding her risk of a recurrent haemorrhage and a planned delivery was discussed. She subsequently presented at 15 weeks' gestation. Following an uneventful pregnancy, she was covered with prophylactic desmopressin and tranexamic acid before a planned induction of labour. She had a normal delivery but placenta was retained. In theatre, an uncomplicated manual removal was followed by massive haemorrhage secondary to uterine atony. Aggressive medical management and B lynch sutures at laparotomy failed to contract the uterus. Hysterectomy was therefore performed. Placental histology later showed evidence of partial placenta diffusa.

**Conclusion:**

Post partum haemorrhage continues to be a leading cause of maternal morbidity and mortality. In this patient, despite identification and attempts at correction of an identified clotting disorder, major obstetric haemorrhage was not avoided. An additional rare placental abnormality was later found. This case highlights the need for medical staff to be aware and alert to unusual risk factors. However, these factors may be unavoidable and early surgical intervention as per local protocol is recommended to minimise maternal morbidity.

## Introduction

Uterine atony is the most common cause of primary post partum haemorrhage. We report a case where this was complicated by two rare conditions, platelet storage pool disease and placenta diffusa. Platelet storage pool disease is a platelet aggregation disorder associated with mild to moderate bleeding diathesis. There are limited cases reported in pregnancy. Placenta diffusa is a rare anomaly where all or part of the fetal membranes remain covered by chorionic villi, and is associated with post partum haemorrhage.

## Case presentation

A 37-year-old Caucasian woman was referred to the obstetric haematology clinic for preconception counselling following a history of three severe post partum haemorrhages. Two of these were complicated by placental retention, requiring manual removal. Investigations including coagulation profile were carried out, which revealed a normal platelet count (192 × 10^9^/litre) and normal bleeding time (8 minutes). A platelet aggregation study showed aggregation defects in response to adrenaline, low concentration (2.5 μM) ADP and low concentration (0.50 mg/ml and 0.75 mg/ml) ristocetin. These laboratory findings were consistent with a diagnosis of platelet storage pool disorder (SPD). Other family members were subsequently investigated. Two of her children were affected by the disorder, suggesting an autosomal dominant pattern of inheritance. She was keen to have another child and was counselled fully regarding the risk of another retained placenta, significant post partum haemorrhage and the possibility of a hysterectomy. A planned delivery was advised, and the risk of her child acquiring the disorder discussed.

She booked at 15 weeks' gestation and a detailed plan for delivery was discussed and documented. Her anomaly scan revealed a singleton pregnancy with no structural abnormalities and a normally sited posterior placenta. Following an uneventful antepartum period, she was admitted at 38 weeks' gestation for induction of labour. In preparation, two units of packed red cells and four units of HLA matched platelets were cross-matched. Before induction, she was given 0.3 μg/kg of prophylactic DDAVP (synthetic desmopressin, deamino-8-D-arginine vasopressin), and 1 g of tranexamic acid.

Labour was induced on the ward with prostaglandin gel and later required augmentation with syntocinon. Following a prolonged first (10 hours 45 minutes) and second stage (1 hour 27 minutes) of labour, she had a normal delivery of a live female infant weighing 3360 g. Despite a bolus of intravenous syntocinon, third stage was not completed after 15 minutes, and she was transferred to theatre without delay.

**Figure 1:**
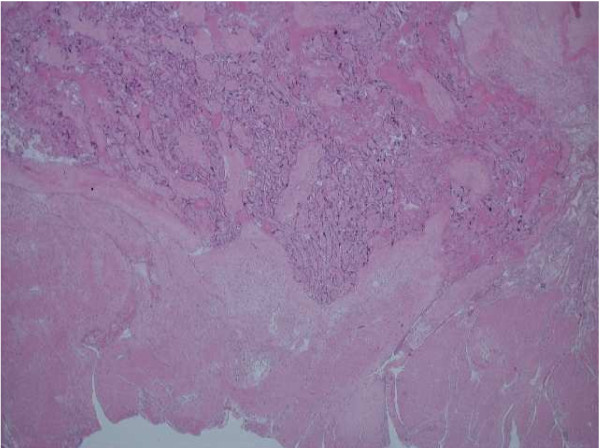
**Microscopic examination demonstrating partial placenta diffusa**. Residual placental tissue was found in seven out of 10 of the blocks randomly sampled from different areas of the uterus, suggesting a wider implantation of placental tissue (partial placenta diffusa) other than the placental disc received separately in the laboratory.

**Figure 2:**
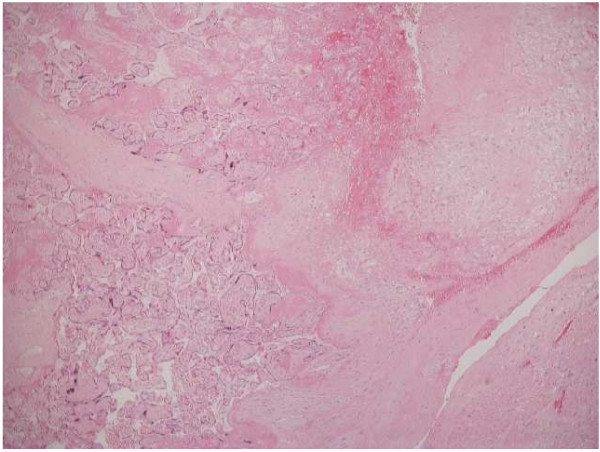
**Higher power image demonstrating the underlying decidua**. The presence of decidua excludes the possibility of placenta creta variants.

An uncomplicated manual removal of placenta was followed immediately by a massive post partum haemorrhage (PPH) secondary to uterine atony. Continuous bimanual compression of the uterus was combined with intravenous oxytocin infusion, intramuscular ergometrine, intramuscular haemabate™ and per rectal misoprostol. These were unsuccessful in achieving adequate uterine tone and a Rusch balloon was inserted into the cavity. This allowed the anaesthetic staff time to stabilise the patient before surgery. At laparotomy, several modified B-lynch sutures were inserted, although the bleeding was reduced, the uterus did not sufficiently contract to maintain haemostasis and a hysterectomy was therefore performed. Total estimated blood loss was 8 litres and the patient required 14 units of packed red cells, 7 platelets, 8 fresh frozen plasmas and 4 units of cryoprecipitate. Post operatively, the patient was transferred to the intensive care unit where she subsequently made a good recovery. Histological analysis of the uterus suggested a wide area of implantation of placental tissue within the uterus, consistent with a diagnosis of partial placenta diffusa or membranacea (Figures 1 and 2).

## Discussion

Post partum haemorrhage continues to be one the leading causes of maternal morbidity and mortality in both developing and developed countries. In this patient, haemorrhage due to uterine atony was complicated by an underlying coagulation disorder and a rare placental abnormality resulting in retained placenta.

Although major PPH can be treated successfully with conservative medical and surgical interventions, in many cases hysterectomy cannot be avoided. The last Confidential Enquiry into maternal deaths identified the possible role of selective arterial embolisation in reducing maternal mortality [1], although it is not always practical to access interventional radiology services in an emergency situation. Recent studies also suggest that treatment with recombinant factor VIIa (NovoSeven), 90 μg/kg, in life-threatening PPH is of benefit. This is an off licence use for the drug, but is now recommended before hysterectomy [2]. Although there has been no particular research in patients with SPD, NovoSeven has been used successfully in patients with severe bleeding secondary to platelet defects such as Glanzmann's thrombasthenia (GT) [3].

Storage pool disorder is a rare platelet abnormality resulting in a moderate bleeding disorder. It comprises a range of disorders whereby platelet dense granules (alpha, delta or both) or their contents, are deficient to variable degrees. Classically, the clinical manifestations include epistaxis, menorrhagia, easy bruising, recurrent anaemia and obstetric or surgical bleeding. The defect may be inherited in isolation or as part of several other congenital syndromes including Wiscott-Aldrich, Chediak-Higachi, syndrome of thrombocytopenia with absent radii and Hermansky-Pudlak syndrome [4]. The inheritance pattern is thought to be autosomal dominant, although penetrance may be variable. Diagnosis is based on laboratory findings including characteristic platelet morphology on peripheral blood smear by electron microscopy, prolonged bleeding time and abnormal platelet aggregation studies. Diagnosis can also be made by flow cytometry, which is used to detect a reduction in mepacrine labelled granules in affected platelets [5].

There have been a limited number of reported cases of pregnancy in patients with platelet storage pool disease [6-8]. In the antenatal period, no intervention is usually necessary; however, a planned delivery is essential. There is no contraindication to vaginal delivery in the absence of other obstetric complications. In labour, fetal scalp electrode, fetal blood sampling and instrumental delivery should be avoided as the baby has a 50% chance of inheriting the disorder. Regional anaesthesia is contraindicated. Labour is covered with DDAVP and although the precise mechanism of action in storage pool disease is unclear, it is thought to stimulate the release of von Willebrand factor (vWf) and thus reduce bleeding time by improving platelet adhesiveness. There is also some evidence supporting the use of antifibrinolytic agents such as tranexamic acid [9]. Although prophylactic DDAVP is potentially life saving in patients with storage pool disorder, a positive response cannot be guaranteed. Blood and HLA matched platelets should therefore be available.

Despite prior knowledge of, and our attempts to manage this patient's platelet disorder, a massive PPH following manual removal of the placenta was not prevented. Placental retention complicates about 3% of all vaginal deliveries. A previous history increases the risk of recurrence by up to three times [10]. Rarely, this can result from abnormal placental implantation and is often diagnosed when there is significant difficulty in separating and removing the placenta. In our patient, manual removal of placenta was uncomplicated but interestingly, histology showed evidence of a very rare placental abnormality known as partial placenta diffusa or membranacea. Although two of her previous deliveries were also complicated by retained placentas, we do not know whether this may have been secondary to placental pathologies, as histological analysis had not been requested. Placenta diffusa is in fact the normal form of placenta, throughout gestation, in several animals such as pigs, donkeys and elephants [11]. In humans, this occurs as a result of abnormal placental development, so that most or all of the fetal membranes remain covered by chorionic villi. Diagnosis by ultrasound is difficult and clinical presentation is varied. It is associated with antepartum haemorrhage (APH), PPH, chorioamnionitis and placental retention. Although half the reported cases have been associated with a live birth, often the outcome is poor, resulting in growth restriction, preterm birth or stillbirths [12].

## Conclusion

Early identification of risk factors for obstetric haemorrhage is vital to the improvement in maternal mortality and morbidity. In our patient, despite preconceptual diagnosis, a planned delivery and attempts at correction of an uncommon underlying coagulopathy, a massive atonic haemorrhage and hysterectomy could not be avoided. In addition to these anticipated risk factors, a rare, unpredictable placental abnormality put this patient at further risk of bleeding. This case highlights the need for medical staff to be alert to rare and unusual risk factors for massive obstetric haemorrhage and the need for multidisciplinary planning to minimise bleeding. However, these additional factors may only exacerbate common life-threatening obstetric situations which may not be avoidable and must be managed according to locally agreed protocols.

## Consent

Written informed consent was obtained from the patient for publication of this case report and any accompanying images. A copy of the written consent is available for review by the Editor-in-Chief of this journal.

## Competing interests

The authors declare that they have no competing interests.

## Authors' contributions

SR, JM and JG prepared the case report and wrote the manuscript. RF performed the histopathology. TJ advised and assisted with the manuscript. All authors read and approved the final manuscript.
